# Supplementary material for: Effects of Perilla Seed Meal on Productive Performance, Egg Quality, Antioxidant Capacity and Hepatic Lipid Metabolism of Wenchang Breeder Hens
Source: Animals (Basel). 2023 Nov 20;13(22):3587. doi: 10.3390/ani13223587 (PMC10668772; doi:10.3390/ani13223587)
Supplement: Supplementary file 1 [file animals-13-03587-s001.zip › animals-2699233-supplementary.pdf]

Supplementary Information

Table S1. The nutritive composition and active ingredients of PSM.

Nutritive ingredients	Content
Crude protein, %	39.75
Crude fat, %	14.87
Crude fiber, %	26.98
Ash, %	5.83
Essential amino acids, %	9.94
Total amino acids, %	32.46
Potassium, mg/kg	9222.33
Ferrous, mg/kg	131.83
**Fatty acid content of PSM**	
C14:0	45.9
C15:0	23.9
C16:0	11706.7
C17:0	39.5
C18:0	3083.3
C20:0	320.0
C22:0	75.7
C24:0	133.3
SFA	15428.3
C16:1	313.3
C17:1	21.2
C18:1n9c	21423.3
MUFA	21757.8
C18:2n6c	16843.3
C20:2	46.0
C18:3n3	63120.0
PUFA	80009.3
